# Ferroptosis in necrotizing enterocolitis: iron overload–driven intestinal injury and mechanistic insights

**DOI:** 10.3389/fped.2026.1873503

**Published:** 2026-07-24

**Authors:** Qingmei Huang, Lingdong Zeng, Bingmei Wei, Qiaozhen Wei, Ruishan Li, Lixue Qin, Qing Chen, Yujun Chen

**Affiliations:** Department of Pediatrics, The Second Affiliated Hospital of Guangxi Medical University, Nanning, China

**Keywords:** ferroptosis, intestinal barrier, iron overload, lipid peroxidation, necrotizing enterocolitis, oxidative stress, preterm infants

## Abstract

Necrotizing enterocolitis (NEC) is a devastating gastrointestinal emergency that primarily affects preterm infants and remains associated with high mortality and significant long-term morbidity. Despite decades of research, the precise mechanisms underlying NEC pathogenesis remain incompletely understood, and effective targeted preventive or therapeutic strategies are limited. Ferroptosis, a regulated form of cell death driven by iron-dependent lipid peroxidation, has recently emerged as an important contributor to intestinal epithelial injury in NEC. Preterm infants are particularly vulnerable to ferroptosis due to immature iron homeostasis, frequent exposure to exogenous iron through supplementation and blood transfusion, heightened oxidative stress, and insufficient antioxidant capacity. Accumulating experimental evidence indicates that iron overload promotes excessive lipid peroxidation, glutathione depletion, and inactivation of key antioxidant enzymes, leading to ferroptotic death of intestinal epithelial cells. This process compromises intestinal barrier integrity, amplifies inflammatory signaling, and interacts with gut microbiota dysbiosis, thereby accelerating NEC progression. In this review, we summarize current advances in understanding the role of ferroptosis in NEC, with an emphasis on iron overload as a central upstream driver and ferroptosis as a convergent execution pathway linking multiple pathogenic factors. We discuss evidence from experimental and translational studies, explore the crosstalk between ferroptosis, inflammation, and the gut microbiota, and outline potential therapeutic implications of targeting ferroptosis. A ferroptosis-centered framework may provide novel insights into NEC pathogenesis and guide future mechanistic and translational research.

## 1 Introduction

Necrotizing enterocolitis (NEC) is one of the most severe gastrointestinal diseases in neonates, predominantly affecting preterm and very low birth weight infants (1–4). The disease is characterized by acute intestinal inflammation, epithelial necrosis, and, in severe cases, intestinal perforation and systemic sepsis (2, 5–9). Despite improvements in neonatal intensive care, the incidence of NEC has remained largely unchanged, with mortality rates approaching 20%–30% in severe cases (2). Survivors frequently experience long-term complications, including short bowel syndrome, growth failure, and neurodevelopmental impairment (10–13).

The pathogenesis of NEC is multifactorial and incompletely understood. Proposed mechanisms include intestinal immaturity, dysbiosis of the gut microbiota, exaggerated inflammatory responses, impaired mucosal perfusion, and oxidative stress (14–16). Multiple forms of programmed cell death—such as apoptosis, necroptosis, and pyroptosis—have been implicated in intestinal epithelial injury during NEC (17–19). However, these mechanisms do not fully explain the pronounced iron-dependent oxidative damage consistently observed in experimental models and human NEC tissues.

Iron overload has emerged as an important but underrecognized contributor to neonatal intestinal injury (20–24). Preterm infants are uniquely susceptible to iron-mediated toxicity because of immature intestinal barrier function, limited antioxidant capacity, and frequent clinical exposure to iron through enteral supplementation and red blood cell transfusion (25–28). Excess iron availability exacerbates oxidative stress, disrupts epithelial integrity, alters gut microbial composition, and amplifies inflammatory responses (21, 28, 29).

Ferroptosis is a distinct form of regulated cell death driven by iron-dependent lipid peroxidation and catastrophic membrane damage (30). Increasing evidence supports a critical role for ferroptosis in NEC-associated intestinal injury, positioning it as a final common execution pathway through which iron overload, oxidative stress, inflammation, and microbial dysbiosis converge to drive epithelial destruction (20–24, 31, 32). This review integrates mechanistic, experimental, and clinical evidence to highlight ferroptosis as a central pathological process in NEC and to discuss its implications for prevention and management.

Literature for this narrative review was identified through searches of PubMed, Web of Science, and Google Scholar, supplemented by manual screening of references from relevant articles. The reviewed literature spans foundational studies on NEC and ferroptosis as well as recent advances published up to March 2026. Key search terms included “ferroptosis”, “necrotizing enterocolitis”, “NEC”, “iron overload”, “iron metabolism”, “oxidative stress”, “lipid peroxidation”, “intestinal barrier”, “gut microbiota”, and “preterm infants”. Particular emphasis was placed on experimental studies, translational research, and clinically relevant publications addressing the role of ferroptosis in NEC pathogenesis and potential therapeutic implications.

## 2 Pathophysiological features of NEC predisposing to ferroptosis

### 2.1 Intestinal immaturity and oxidative vulnerability

The preterm intestine is structurally and functionally immature, characterized by incomplete epithelial differentiation, impaired tight junction assembly, and underdeveloped mucosal defenses (6, 33–35). This intrinsic fragility is exacerbated by immature antioxidant systems, particularly the glutathione (GSH) redox pathway, which limits the capacity to detoxify reactive oxygen species (ROS) in preterm infants (36). The imbalance between ROS production and insufficient antioxidant defenses creates a permissive environment for lipid peroxidation and ferroptotic cell death, thereby increasing susceptibility to NEC (20, 22, 23, 32, 36–41).

### 2.2 Inflammation and immune dysregulation

NEC is a severe inflammatory intestinal disease primarily affecting preterm infants, characterized by exaggerated innate immune activation and impaired immunoregulatory mechanisms (42). An important feature in NEC pathogenesis is the infiltration of inflammatory macrophages into the intestinal mucosa, which release proinflammatory cytokines (22, 43, 44). This inflammatory milieu significantly disrupts iron homeostasis, leading to increased intracellular iron availability and suppressed iron export within intestinal cells (45). Concurrently, regulatory T cell (Treg) depletion further exacerbates this inflammatory injury (41, 46, 47). This combination of inflammatory activation, impaired immune regulation and iron dysregulation may increase the susceptibility of intestinal epithelial cells to ferroptosis, thereby promoting oxidative stress and tissue damage. Collectively, these processes may establish a feed-forward loop between ferroptosis and intestinal inflammation that contributes to NEC progression (24, 41, 45).

### 2.3 Ischemia–reperfusion injury and mitochondrial dysfunction

Ischemia-reperfusion injury (IRI) is a significant contributor to the pathogenesis of NEC (42, 48–50). This complex process involves an initial period of hypoxia followed by reoxygenation, which collectively induces substantial mitochondrial dysfunction and excessive production of ROS (50–52). The resulting cellular damage is associated with iron-dependent oxidative injury and increased susceptibility to ferroptosis, a form of regulated cell death increasingly linked to NEC pathogenesis (37, 53).

## 3 Molecular machinery of ferroptosis in NEC

Ferroptosis is a form of regulated cell death characterized by iron-dependent lipid peroxidation and has emerged as a potentially important contributor to the pathogenesis of NEC, a severe gastrointestinal condition primarily affecting premature infants (18, 54). Current evidence from experimental NEC models and limited human studies suggests that ferroptosis may contribute to intestinal epithelial injury and barrier dysfunction in NEC (22, 23, 40, 55). The molecular machinery underlying ferroptosis in NEC involves a complex interplay between iron availability, lipid peroxidation, and the cell's antioxidant defense systems, notably glutathione peroxidase 4 (GPX4) and ferroptosis suppressor protein 1 (FSP1) (20, 54).

### 3.1 GPX4 and glutathione depletion

GPX4 is a central enzyme crucial for detoxifying lipid hydroperoxides and preventing ferroptosis (20, 54, 56). GPX4 utilizes GSH as a cofactor to reduce lipid hydroperoxides to less harmful lipid alcohols, thereby maintaining cellular redox balance (20, 56–58). In NEC, a notable decline in the number of regulatory T cells (Tregs) within intestinal tissues contributes to excessive inflammation and necrosis, with ferroptosis of these cells being a focus of investigation (41). Studies have identified a deficiency in GPX4 and an accumulation of lipid radicals in NEC, highlighting the enzyme's critical role in this disease (20).

The synthesis of GSH depends on cystine uptake via system Xc^−^, whose functional subunit is solute carrier family 7 member 11 (SLC7A11) (59). Impaired cystine uptake due to SLC7A11 dysfunction leads to reduced GSH synthesis, thus compromising GPX4 activity and exacerbating ferroptotic susceptibility (59, 60). N-acetyl-L-cysteine (NAC), a precursor of GSH, is a well-established inhibitor of ferroptosis by replenishing intracellular GSH and preserving GPX4 function, thereby limiting lipid peroxidation (58). In experimental NEC models,NAC has been reported to inhibit ferroptosis in intestinal epithelial cells partly through downregulation of SESN2 expression, thereby alleviating intestinal injury (40, 58). NAC and its enantiomer N-acetyl-d-cysteine (d-NAC) have been identified as direct reducing substrates of GPX4, indicating that GPX4 may utilize alternative reducing substrates to counteract ferroptosis when glutathione is deficient (58). Evidence from nephritic mouse models has similarly demonstrated reduced SLC7A11 expression, impaired glutathione synthesis, and decreased GPX4 levels (61). Although obtained from a non-NEC disease model, these findings are consistent with the concept that disruption of the SLC7A11–GSH–GPX4 axis promotes ferroptosis susceptibility.

### 3.2 Lipid peroxidation and ACSL4

Lipid peroxidation, the oxidative degradation of lipids, is a hallmark of ferroptosis, leading to membrane damage and cell death (54). Acyl-CoA synthetase long-chain family member 4 (ACSL4) plays a critical role in promoting this process (55, 62). ACSL4 is responsible for incorporating polyunsaturated fatty acids (PUFAs) into membrane phospholipids, making the membranes highly susceptible to peroxidation (54). Upregulation of ACSL4 in NEC tissues correlates positively with increased lipid peroxidation and ferroptotic injury (55). Inhibition of ferroptosis significantly alleviates NEC in newborn mice, and ACSL4 expression levels are augmented and positively correlated with ferroptosis in NEC.

The ACSL4-LPCAT3 axis acts as a crucial lipid remodeling pathway that supplies phospholipid substrates for ferroptosis (62, 63). For example, a lactoferrin-derived peptide, LFDP1, has been shown to alleviate experimental NEC by blocking the ACSL4-LPCAT3 axis, which helps reduce ferroptosis (63). Ferroptosis triggered by the STAT1-IRF1-ACSL4 pathway has also been implicated in radiation-induced intestinal injury (64). Although not derived from NEC models, these findings further support the importance of ACSL4-mediated lipid remodeling in intestinal ferroptosis. Given its central role in ferroptosis regulation, ACSL4 has emerged as a potential therapeutic target for ferroptosis-related diseases, and several ACSL4-targeting compounds have demonstrated anti-ferroptotic activity in experimental settings (65).

### 3.3 GPX4-independent defense mechanisms

While the GPX4-glutathione axis is a primary defense against ferroptosis, cells also possess GPX4-independent mechanisms to combat lipid peroxidation (66). FSP1 (also known as AIFM2) is a crucial component of such a pathway (66, 67). FSP1 acts by regenerating reduced coenzyme Q10 (CoQ10) from its oxidized form (ubiquinone) using NAD(P)H as an electron donor (66, 68). The reduced CoQ10, or ubiquinol, then functions as a lipophilic antioxidant, directly scavenging lipid radicals and preventing the propagation of lipid peroxidation within cell membranes (68). This FSP1-CoQ10-NAD(P)H axis provides a parallel and independent defense system that protects cells from ferroptotic injury when GPX4 activity is compromised (66). Although direct evidence regarding FSP1-mediated ferroptosis regulation in NEC remains limited, studies in other ferroptosis-related diseases have highlighted the importance of the FSP1–CoQ10 pathway in maintaining cellular redox homeostasis and resistance to ferroptosis (66, 68). For example, neuron-targeted liposomal CoQ10 has been shown to attenuate neuronal ferroptosis after subarachnoid hemorrhage by activating the FSP1/CoQ10 system (68). These findings provide mechanistic insights that may be relevant to NEC. Future studies are needed to determine whether the GPX4-independent FSP1–CoQ10 pathway contributes to ferroptosis regulation during NEC.

## 4 Iron overload as an upstream driver of ferroptosis

### 4.1 Clinical iron exposure and expansion of the labile iron pool

Preterm infants are highly susceptible to iron imbalance due to various factors including a shorter gestation period, rapid postnatal growth, and frequent medical interventions (28). Enteral iron supplementation is a common practice in preterm and low-birth-weight infants to prevent iron deficiency anemia, which can severely impact growth and neurodevelopment (69, 70). However, the immature iron regulatory mechanisms in these infants mean that supplementation, while essential, carries the risk of iron overload (71). The debate continues regarding the optimal dosage to ensure adequate iron for development without inducing toxicity. Red blood cell transfusions are frequently administered to preterm infants to manage anemia, and repeated transfusions may substantially increase the circulating iron burden because each unit of packed red blood cells contains a considerable amount of iron (72, 73). Similarly, hemolysis, the breakdown of red blood cells, whether endogenous or transfusion-related, releases heme and iron into circulation (37). This increased iron load can overwhelm the body's capacity to safely sequester iron, leading to an expansion of the labile iron pool(LIP) (28, 37). Under inflammatory conditions, such as those characteristic of NEC, the management of intracellular iron is further disrupted. Expansion of the LIP is considered a key biochemical event linking clinical iron exposure to increased ferroptosis susceptibility. Excess labile iron promotes Fenton chemistry, leading to ROS accumulation, lipid peroxidation, progressive cellular dysfunction, which may contribute to ferroptotic cell death under pathological conditions (74, 75). Although direct quantification of the labile iron pool in human NEC tissues remains limited, experimental studies support a close association between iron overload, oxidative stress, and ferroptosis-related intestinal injury.

### 4.2 Iron-catalyzed oxidative stress and epithelial injury

Excess ferrous iron is a key driver of ferroptosis, a form of regulated cell death characterized by iron-dependent lipid peroxidation and membrane damage (76). Excess ferrous iron is a key driver of ferroptosis, a distinct form of regulated cell death characterized by iron-dependent lipid peroxidation and membrane damage (76). Intestinal hypoxia and iron overload have been implicated as important contributors to ferroptosis-associated intestinal injury (29). For instance, an iron overload mouse model established by intraperitoneal injection of 120 mg/kg body weight iron dextran every fortnight led to observable oxidative stress and intestinal damage (77). Similarly, high iron diets have been shown to induce colitis by modulating ferroptosis and interfering with gut microbiota in mice (29). Although these findings were not derived from NEC models, they provide mechanistic evidence linking iron overload to intestinal ferroptosis and epithelial injury. Excess oral iron in the intestinal tract produces reactive oxygen species via Fenton and Haber-Weiss reactions, triggering oxidative stress and lipid peroxidation, which in turn induces ferroptosis, apoptosis, and necrosis (78). Collectively, these findings support a mechanistic link between iron overload, oxidative stress, lipid peroxidation, and ferroptosis-related intestinal injury (78, 79).

### 4.3 Interactions with the gut microbiota

Iron availability significantly influences the intestinal microbial ecosystem, and disruptions in this delicate balance, particularly involving excess luminal iron, are increasingly recognized as critical factors in the pathogenesis of NEC in premature infants (21, 24, 80).

Excess luminal iron may alter gut microbial composition by promoting the expansion of certain opportunistic bacteria, such as Clostridium species, while reducing beneficial commensals, particularly Bifidobacterium and Lactobacillus, thereby contributing to gut dysbiosis (81, 82). These alterations may reflect the differential responses of intestinal microorganisms to increased luminal iron availability, favoring the growth of some iron-responsive bacteria while disadvantaging beneficial commensals. This dysbiotic state may contributes to intestinal inflammation, oxidative stress, and increased susceptibility to ferroptosis, thereby creating a microenvironment that favors ferroptosis and accelerates NEC progression (20, 22, 24, 37, 55, 83). Although much of the mechanistic evidence linking iron overload, gut dysbiosis, and ferroptosis has been derived from experimental models and related intestinal disorders, these findings provide important insights into how iron-mediated microbial alterations may contribute to NEC pathogenesis.

**Figure 1:**
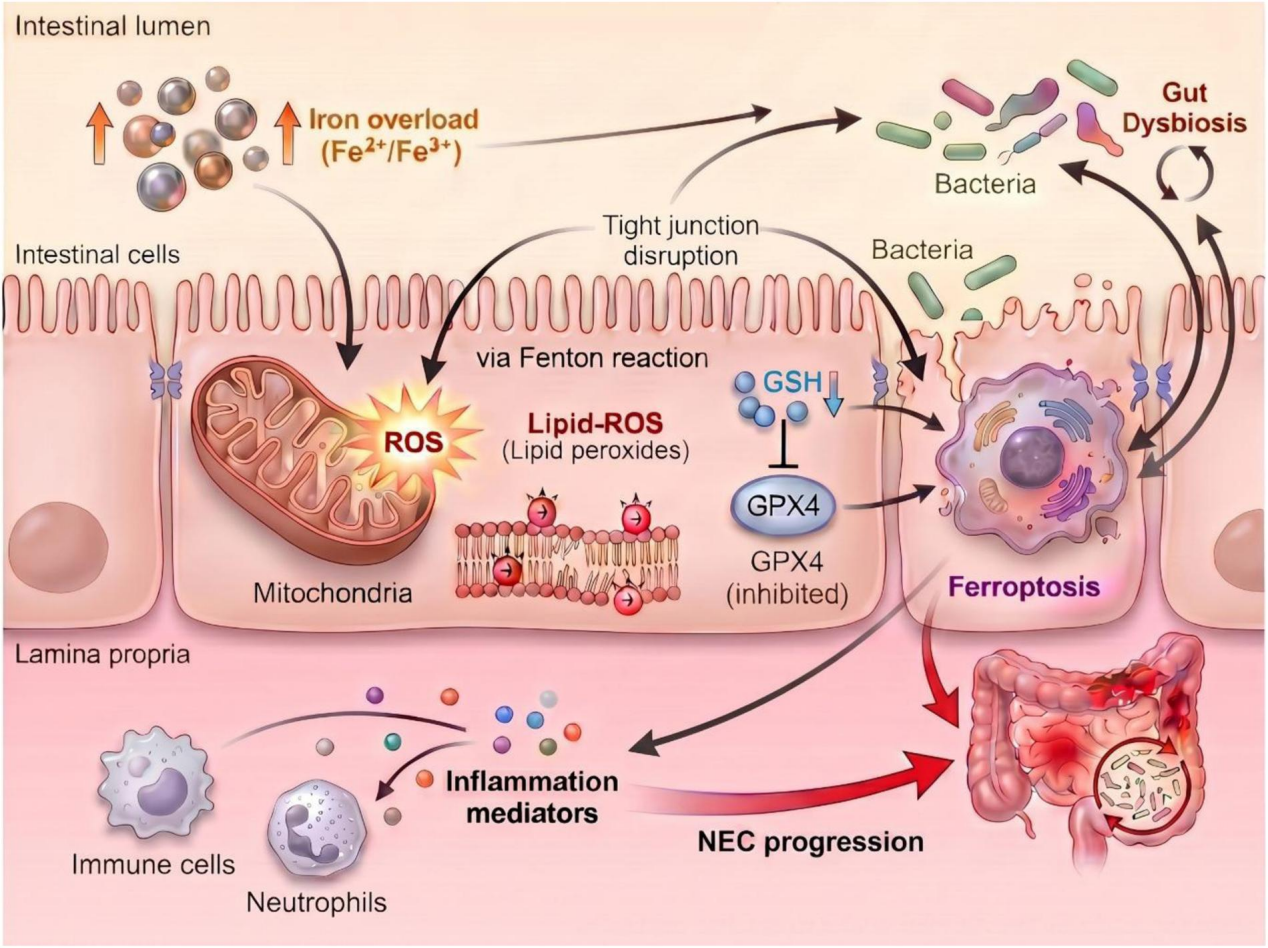
Proposed role of iron overload–driven ferroptosis in the pathogenesis of necrotizing enterocolitis (NEC). Excess luminal and intracellular iron promotes reactive oxygen species (ROS) generation through Fenton reactions, leading to lipid peroxidation, depletion of glutathione (GSH), inhibition of glutathione peroxidase 4 (GPX4), and ferroptotic cell death in intestinal epithelial cells. Ferroptosis may contribute to intestinal barrier dysfunction, inflammatory responses, and gut microbiota dysbiosis, thereby potentially promoting NEC progression. The figure illustrates a proposed mechanistic framework based on current experimental and translational evidence. NEC, necrotizing enterocolitis; ROS, reactive oxygen species; GSH, glutathione; GPX4, glutathione peroxidase 4.

Collectively, current evidence suggests that iron overload may represent an important upstream factor linking microbial dysbiosis, oxidative stress, and ferroptosis-associated intestinal injury in NEC (Figure 1).

## 5 Experimental and human evidence supporting ferroptosis in NEC

NEC is a devastating gastrointestinal disease in preterm infants characterized by severe intestinal inflammation and necrosis (20). Emerging evidence increasingly supports the involvement of ferroptosis, a distinct form of iron-dependent regulated cell death driven by lipid peroxidation, in the pathogenesis of NEC (22–24, 55). Animal models of NEC consistently exhibit hallmark features of ferroptosis, including GPX4 downregulation, ACSL4 upregulation, glutathione depletion, and accumulation of lipid peroxidation products such as malondialdehyde and 4-hydroxynonenal (20, 36, 41, 55). Pharmacological inhibition of ferroptosis using various agents has shown marked protective effects in NEC animal models. Ferrostatin-1 (Fer-1), a potent ferroptosis inhibitor, has been shown to protect against NEC-associated intestinal injury in mouse pups (23). It significantly improved survival and reduced injury by inhibiting lipid peroxidation. Similarly, liproxstatin-1, another radical-trapping antioxidant, has been recognized for its ability to suppress ferroptosis by inhibiting lipid peroxidation (84). Iron chelators, such as deferoxamine, sequester catalytic iron, thereby preventing iron-dependent lipid peroxidation and subsequent ferroptosis (84). The efficacy of these inhibitors in ameliorating intestinal injury and improving survival in NEC models strongly supports the mechanistic role of ferroptosis in this disease.

Human studies further corroborate these findings, emphasizing the translational relevance of ferroptosis in human NEC. Analyses of resected intestinal tissues from infants with NEC reveal altered expression of ferroptosis-related genes and proteins (20, 55). For example, studies have observed a deficiency in GPX4 in human intestinal tissue from NEC patients (20). Transcriptomic data from NEC samples indicate an enrichment of ferroptosis-associated pathways (22). Furthermore, increased levels of lipid peroxidation, as indicated by markers like MDA and 4-HNE, in human NEC tissues correlate with disease severity (20, 36). These human translational data support the involvement of ferroptosis in NEC and suggest that ferroptosis may represent a clinically relevant mechanism in affected infants (20, 22, 55). However, it should be noted that several ferroptosis-associated markers, including lipid peroxidation products and antioxidant pathway alterations, may also be observed in other forms of severe intestinal inflammation, oxidative stress, and tissue injury. Markers such as MDA and 4-HNE are therefore not specific indicators of ferroptosis, whereas alterations in GPX4, ACSL4, and lipid-ROS provide stronger mechanistic evidence. Therefore, while current human data support the involvement of ferroptosis in NEC, the specificity of these markers and their causal contribution to disease progression require further investigation. Future studies integrating functional assessment of the FSP1 pathway, profiling of ferroptosis-specific oxidized phospholipids, and cell type-specific analyses may provide more direct evidence to strengthen the causal role of ferroptosis in NEC.

## 6 Crosstalk between ferroptosis and intestinal inflammation

Ferroptosis, an iron-dependent cell death, fuels inflammation in the immature intestine, creating a feed-forward loop in conditions such as NEC (83, 85–87). This process is characterized by iron toxicity, lipid peroxidation, and plasma membrane damage, leading to the release of pro-inflammatory mediators (75, 85, 87, 88). The rupture of ferroptotic intestinal epithelial cells (IECs) in NEC leads to the release of specific damage-associated molecular patterns (DAMPs), including oxidized phospholipids and high-mobility group box 1 (HMGB1) (86, 87, 89–91). These mediators are critical for activating innate immune pathways and amplifying local inflammatory responses (86, 89, 91). Among these mediators, HMGB1, a representative DAMP, has been shown to mediate inflammation and exacerbate intestinal barrier injury in ulcerative colitis by promoting ferroptosis through TLR4/NF-*κ*B signaling and GPX4 suppression (92, 93). Although these findings were not derived from NEC models, they provide mechanistic insights into the interplay between ferroptosis and intestinal inflammation. Emerging evidence further suggests that ferroptosis in immune cells, particularly regulatory T cells and macrophages, may also contribute to immune dysregulation and intestinal inflammation in NEC, although direct evidence in NEC remains limited (22, 41). DAMP sensing triggers inflammation, which can lead to dysregulated inflammatory diseases, further disrupting iron homeostasis and antioxidant capacity (91, 94, 95). Increased inflammation expands the labile iron pool, promoting ferroptosis through free radical generation and lipid peroxidation (75, 76, 86, 95). Inflammation-related signaling pathways, including NF-*κ*B, have been implicated in the regulation of ferroptosis, suggesting a potential molecular link between inflammatory signaling and ferroptotic cell death (86, 96).

Evidence from other disease models suggests that ferroptosis may interact with other forms of regulated cell death, including pyroptosis and necroptosis (54, 75, 97–99). Ferroptosis-associated ROS have been implicated in the activation of the NLRP3 inflammasome, whereas ferroptosis and necroptosis share common features, including membrane disruption, DAMP release, and amplification of inflammatory responses (54, 97–99). Although these interactions have not yet been characterized in NEC, they underscore the need for future studies to clarify the interplay among different regulated cell death pathways during NEC progression.

Collectively, current evidence suggests a reciprocal interaction between ferroptosis and intestinal inflammation, whereby each process may amplify the other, contributing to epithelial barrier dysfunction and disease progression in NEC (18, 22, 24, 32, 38, 40, 55).

## 7 Therapeutic strategies for NEC via ferroptosis modulation

### 7.1 Targeted ferroptosis inhibitors

Given the emerging role of ferroptosis in NEC pathophysiology, targeting this regulated cell death pathway offers promising therapeutic opportunities. To this end, several pharmacological interventions have demonstrated significant efficacy in mitigating intestinal injury. Canonical ferroptosis inhibitors, such as Ferrostatin-1, have been shown to protect the intestinal mucosa in murine NEC models by directly abrogating ferroptotic signaling (23). Similarly, NAC—a biosynthetic precursor of glutathione—augments intracellular GSH pools and preserves GPX4 function, thereby inhibiting ferroptosis (58). In experimental NEC models, NAC has also been reported to suppress ferroptosis in enterocytes through downregulation of SESN2 (40). Furthermore, emerging evidence highlights the therapeutic potential of L-cystine and ALAS2. L-cystine has been shown to alleviate NEC by suppressing ferroptosis in intestinal epithelial cells while restoring Th17/Treg immune balance through inhibition of the IL-6/STAT3 signaling pathway, highlighting a shared IL-6/STAT3-dependent mechanism linking epithelial ferroptosis and immune regulation (38). In addition, ALAS2 has been reported to protect intestinal epithelial cells by suppressing oxidative stress-driven ferroptosis, thereby reducing intestinal injury in experimental NEC models (24). Representative ferroptosis-targeted therapeutic strategies and the current level of evidence supporting their application in NEC are summarized in Table 1. However, most available evidence is derived from preclinical studies, and further investigation is required to determine the safety, optimal timing, dosing strategies, and clinical feasibility of ferroptosis-targeted interventions in preterm infants.

**Table 1:** Summary of therapeutic strategies targeting ferroptosis in NEC.

Strategy category	Representative agent(s)	Evidence in NEC	Representative references
Targeted ferroptosis inhibitors	Ferrostatin-1 (Fer-1)	Human intestinal organoid and animal studies	(23)
	N-acetyl-L-cysteine (NAC)	*In vitro* and animal studies	(40)
	L-cystine	*In vitro* and animal studies	(38)
	5′-aminolevulinate synthase 2 (ALAS2)	*In vitro* and animal studies	(24)
Iron chelation	Deferoxamine	No direct evidence in NEC	(21)
Antioxidants	Vitamin E (*α*-tocopherol)	*In vitro* and animal studies	(41)
	Coenzyme Q10 (CoQ10)	No direct evidence in NEC	(101, 102)
Nrf2/GPX4 activation	Nrf2 activators	No direct evidence in NEC	(103)
Breast milk-based protection	Lactoferrin/LFDP1	*In vitro* and animal studies, clinical studies	(63, 104, 105)
Breast milk–derived exosomes	miR-375-3p	Animal studies	(106)

### 7.2 Iron chelation therapy

Iron chelating agents, exemplified by deferoxamine, function by binding labile intracellular iron to suppress the Fenton reaction, consequently curbing ROS production and lipid peroxidation-driven ferroptosis (21). While reducing excess intracellular iron may represent a promising therapeutic strategy for NEC, the clinical translation of these agents in preterm infants requires careful evaluation of their safety profiles, optimal dosing regimens, and potential effects on erythropoiesis and neurodevelopment to avoid adverse consequences such as systemic iron depletion.

### 7.3 Antioxidant interventions against lipid peroxidation

As lipophilic antioxidants, Vitamin E and its derivatives function by directly neutralizing lipid-derived free radicals, thereby halting the lipid peroxidation chain reaction (100). Emerging evidence indicates that Vitamin E mitigates NEC-associated inflammation by safeguarding Treg cells against ferroptotic cell death (41). Additionally, the endogenous antioxidant CoQ10 serves to counteract lipid peroxidation (101, 102). Although direct evidence supporting CoQ10 and natural flavonoids in NEC remains limited, studies in other ferroptosis-related diseases suggest that these compounds may suppress ferroptosis through their antioxidant properties (101). Their potential therapeutic value in NEC warrants further investigation.

### 7.4 Stabilization of GPX4 and activation of the Nrf2 signaling pathway

Increasing the protein stability or transcriptional expression of GPX4 is a well-established strategy to counteract ferroptosis (20, 101). Nrf2 activators attenuate oxidative stress and cellular damage by promoting GPX4 expression and coordinating downstream antioxidant defense pathway (103). Although direct evidence supporting Nrf2-targeted therapies in NEC remains limited, studies in ferroptosis-related diseases suggest that activation of the Nrf2–GPX4 axis may represent a promising strategy for reducing ferroptosis-associated intestinal injury.

### 7.5 Breast milk–based nutritional protection

Breast milk is widely recognized as an important protective factor against NEC. Studies have shown that lactoferrin supplementation can reduce the severity of NEC by upregulating intestinal epithelial proliferation and modulating immune responses (104, 105). LFDP1 has also been shown to alleviate experimental NEC by blocking the ACSL4-LPCAT3 axis, which is involved in ferroptosis (63). Breast milk-derived exosomes are another crucial component contributing to the anti-ferroptotic and protective effects against NEC. For instance, specific miRNAs within exosomes, such as miR-375-3p, have been implicated in attenuating intestinal injury in NEC by targeting proteins like YWHAB (106). Exosomes also carry enzymes and other molecules that bolster antioxidant defenses and may stabilize GPX4 expression (107). Collectively, these findings suggest that the protective effects of breast milk may be partially mediated through ferroptosis-related pathways.

Collectively, these findings suggest that modulation of ferroptosis represents a promising adjunctive therapeutic avenue in NEC, although further clinical validation is required.

## 8 Limitations of current evidence

Despite increasing evidence supporting the involvement of ferroptosis in NEC, several important limitations should be acknowledged. First, much of the current evidence is derived from animal models and *in vitro* studies, whereas direct evidence from human NEC tissues remains relatively limited. Second, many ferroptosis-associated markers, including lipid peroxidation products and alterations in antioxidant pathways, are not specific to ferroptosis and may also reflect severe inflammation, oxidative stress, or tissue injury. Consequently, establishing a causal role for ferroptosis in NEC based solely on tissue markers remains challenging. Third, ferroptosis interacts extensively with other forms of regulated cell death, including apoptosis, necroptosis, and pyroptosis, and the relative contribution of each pathway to NEC pathogenesis remains incompletely understood. Finally, although several ferroptosis-targeted interventions have demonstrated promising protective effects in experimental NEC models, their safety and clinical applicability in preterm infants require further investigation.

## 9 Conclusions and future directions

Emerging evidence supports the involvement of ferroptosis in NEC and suggests that ferroptosis may represent an important mechanistic link among iron dysregulation, oxidative stress, inflammation, and intestinal barrier dysfunction. By identifying iron overload as a potentially modifiable upstream factor, this review underscores opportunities for prevention and intervention through optimized iron management and targeted nutritional strategies. Future studies should focus on validating biomarkers of iron overload and ferroptosis in human NEC tissues to facilitate early risk stratification and individualized preventive strategies. Further investigations are also needed to clarify cell type-specific ferroptotic mechanisms, particularly in intestinal epithelial cells and immune cell populations, investigate the potential interplay between ferroptosis and other forms of regulated cell death, and evaluate the safety and efficacy of ferroptosis-targeted therapies in preterm infants. Better understanding of the distinct roles of ferroptosis in different cell types may facilitate the development of more precise cell-targeted therapeutic strategies for NEC. Integrating pathway-specific pharmacological inhibitors together with complementary genetic approaches may help distinguish the relative contribution of different regulated cell death pathways during NEC progression. Improved understanding of ferroptosis may facilitate the development of novel preventive and therapeutic approaches for NEC.
